# Isolation of a Δ5 Desaturase Gene from *Euglena gracilis* and Functional Dissection of Its HPGG and HDASH Motifs

**DOI:** 10.1007/s11745-012-3690-1

**Published:** 2012-06-24

**Authors:** Dana Walters Pollak, Michael W. Bostick, Hyeryoung Yoon, Jamie Wang, Dieter H. Hollerbach, Hongxian He, Howard G. Damude, Hongxiang Zhang, Narendra S. Yadav, Seung-Pyo Hong, Pamela Sharpe, Zhixiong Xue, Quinn Zhu

**Affiliations:** Biochemical Sciences and Engineering, Central Research and Development, E. I. du Pont de Nemours and Company, Wilmington, DE 19880 USA

**Keywords:** Δ5 desaturase, HPGG motif, HDASH motif, Double mutant, Fatty acid biosynthesis, *Yarrowia lipolytica*

## Abstract

Delta (Δ) 5 desaturase is a key enzyme for the biosynthesis of health-beneficial long chain polyunsaturated fatty acids such as arachidonic acid (ARA, C20:4n-6), eicosapentaenoic acid (C20:5n-3) and docosahexaenoic acid (C22:6n-3) via the “desaturation and elongation” pathways. A full length Δ5 desaturase gene from *Euglena gracilis* (*EgΔ5D*) was isolated by cloning the products of polymerase chain reaction with degenerate oligonucleotides as primers, followed by 5′ and 3′ rapid amplification of cDNA ends. The whole coding region of *EgΔ5D* was 1,350 nucleotides in length and encoded a polypeptide of 449 amino acids. BlastP search showed that *EgΔ5D* has about 39 % identity with a Δ5 desaturase of *Phaeodactylum tricornutum*. In a genetically modified dihomo-gamma-linoleic acid (DGLA, C20:3n-6) producing *Yarrowia lipolytica* strain, *EgΔ5D* had strong Δ5 desaturase activity with DGLA to ARA conversion of more than 24 %. Functional dissection of its HPGG and HDASH motifs demonstrated that both motifs were important, but not necessary in the exact form as encoded for the enzyme activity of *EgΔ5D.* A double mutant *EgΔ5D*-*34G158G* with altered sequences within both HPGG and HDASH motifs was generated and exhibited Δ5 desaturase activity similar to the wild type *EgΔ5D*. Codon optimization of the N-terminal region of *EgΔ5D*-*34G158G* and substitution of the arginine with serine at residue 347 improved substrate conversion to 27.6 %.

**Electronic supplementary material:**

The online version of this article (doi:10.1007/s11745-012-3690-1) contains supplementary material, which is available to authorized users.

## Introduction

**Fig. 1:**
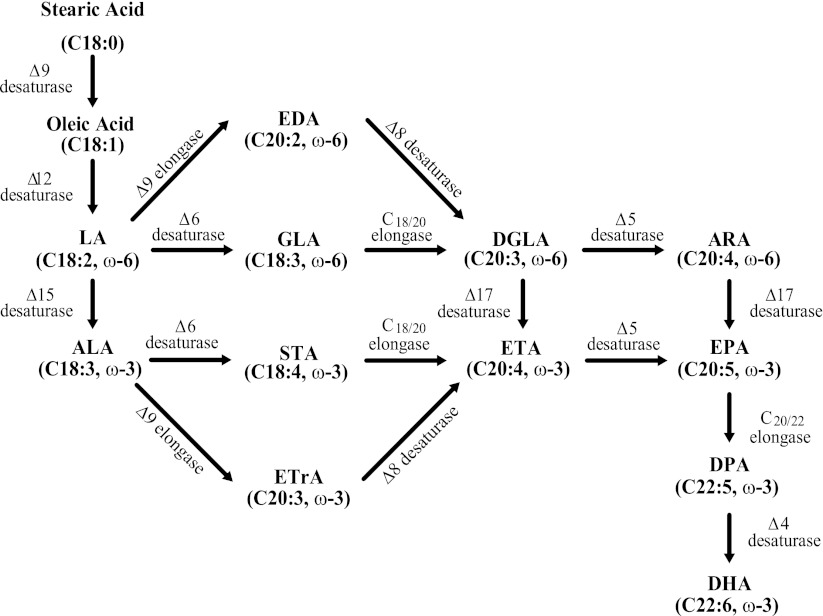
Biosynthetic pathways for ARA, EPA and DHA via “desaturation and elongation” pathways

There is increasing interest in the recognized health benefits of long chain polyunsaturated fatty acids (LC-PUFA) such as arachidonic acid (ARA, C20:4n-6), eicosapentaenoic acid (EPA, C20:5n-3) and docosahexaenoic acid (DHA, C22:6n-3) for both humans and animals [1–3]. Since mammals lack delta (Δ) 12- and Δ15-desaturases, ARA, EPA and DHA cannot be synthesized de novo and must be obtained either in the diet or synthesized through “desaturation and elongation” pathways (Fig. 1) from essential fatty acids linoleic acid (LNA, 18:2n-6) and/or alpha (α)-linolenic acid (ALA 18:3n-3). These LC-PUFA are important fatty acids for human growth and development. For example, ARA, a precursor of EPA, is abundant in the brain and muscles. As a lipid second messenger ARA is involved in cellular signaling and is a key inflammatory intermediate [3]. EPA is a precursor of DHA, and induces a broad anti-inflammatory response [1–3]. DHA is a major ω-3 fatty acid in the mammalian central nervous system and enhances synaptic activities in neuronal cells [1–4]. EPA and DHA are the precursors of E- and D-series resolvins, respectively. These two classes of resolvins have distinct structural, biochemical and pharmacological properties [5, 6]. ARA and DHA together play critical roles for neurological development and health [4, 7]. Dietary EPA and DHA can effectively reduce the level of blood triglycerides in human [8]. Increased intake of EPA-rich supplement has beneficial effects on coronary heart disease, high blood pressure, inflammatory disorders and mental illness [9, 10].

Currently, the primary source of EPA and DHA is marine fish oil. Most of the EPA and DHA in fish oil are from their cold-water oceanic microalgae food sources. More than 85 % of isolated fish oil is used for aquaculture. In the case of salmon-farming, fish oils from approximately 4 pounds of fish are needed to raise one pound of salmon filet, the fish-in and fish-out ratio is about 4:1. In today’s environment, wild-caught fish often contain contaminants such as methylmercury, polychlorinated biphenyls, dioxins and several other halogenated persistent organic pollutants [11]. With ever-growing human populations, and limited sources of ocean fish, there is growing concern about the quality, quantity and sustainability of fish oil.

In the last two decades, great efforts have been focused on developing different hosts for production of LC-PUFA. Wild type *Mortierella alpina* has been developed for commercial production of ARA [12], while *Crypthecodinium cohnii* and *Schizochytrium* have been developed for commercial production of DHA [13]. The ARA and DHA oils produced from these organisms have been largely used in infant formulas. *Yarrowia lipolytica* has been genetically engineered to contain an EPA biosynthesis pathway [14] allowing for the commercial production of EPA oil, NewHarvest™ (http://www.newharvest.com). The EPA oil has been used as a human nutritional supplement. Additionally, EPA-rich *Yarrowia* biomass has been used to feed a brand of farmed salmon, Verlasso™ (http://www.verlasso.com), with a fish-in and fish-out ratio of about 1:1. However, the current production scale and cost of ARA, EPA and DHA cannot meet the market demand.

Various organisms use different pathways to synthesize ARA, EPA and DHA. *Crypthecodinium* and *Schizochytrium* synthesize EPA and DHA through a polyketide-based pathway [15], while some species of algae, fungi, and protists synthesize ARA, EPA and DHA through fatty acid “desaturation and elongation” pathways [16, 17]. All “desaturation and elongation” pathways (Fig. 1) require LNA and/or ALA as a substrate, followed by the “Δ9 elongase and Δ8 desaturase” pathway or the “Δ6 desaturase and C_18/20_ elongase” pathway to synthesize ARA, EPA and DHA by orchestrated elongation and desaturation reactions. So far, genetic engineering has mainly employed these “desaturation and elongation” pathways to modify hosts such as plants and yeast to produce ARA, EPA and DHA [14, 18–23]. Production of ARA, EPA and DHA through “desaturation and elongation” pathways requires gene expression of Δ5 desaturase to catalyze the conversion of di-homo-γ-linolenic acid (DGLA, C20:3n-6) to ARA, with a similar activity of converting eicosatetraenoic acid (ETA, C20:4n-3) to EPA. Since the isolation of Δ5 desaturase gene from *Mortierella alpina * (*MaΔ5D*, 24, 25), several Δ5 desaturase genes have been isolated [26], however, there is little research about their structure/function relationship. Additionally, more efficient Δ5 desaturase genes are needed to ensure engineered organisms may produce high levels of ARA, EPA and DHA.

Δ5 desaturases are known as “front-end” desaturases, wherein desaturation occurs between a pre-existing double bond and the carboxyl terminus of the fatty acid [26–29]. Like other desaturases, Δ5 desaturase is an iron-containing and membrane-bound enzyme that requires both molecular oxygen and an electron transfer to introduce double bonds into an existing acyl chain. Microsomal cytochrome *b*_5_ serves as electron donor to desaturase enzymes [26, 29, 30]. Fatty acid desaturation can be carried out via concerted action of multiple enzymes including NADH reductase, the desaturase enzyme, and cytochrome *b*_5_ reductase. Alternatively, many desaturases contain both a cytochrome *b*_5_ domain and a desaturase domain. For example, Δ4, Δ5, Δ6 and Δ8 desaturases have a cytochrome *b*_5_ domain at their N-terminus [26]; Δ9 desaturase has a cytochrome *b*_5_ domain at its C-terminus [30]. The cytochrome *b*_5_ domain of all desaturases has a heme-binding “HPGG” motif. Previous studies using molecular dynamics simulations suggest the “HPGG” motif is important for heme group assembly and desaturase function [31, 32].

The active site of desaturases has been characterized as a diiron cluster that is bound to the enzyme by three regions of highly conserved histidine-rich (His-rich) motifs [27, 30]. These three His-rich motifs H(X)_3–4_H, H(X)_2–3_HH, and H/Q(X)_2–3_HH are conserved among all front-end desaturases, and the eight histidine residues of these three motifs are essential for catalytic activity [33]. In the case of Δ5 desaturase from *MaΔ5D* [24, 25], the exact amino acid sequence of the first His-rich motif (H(X)_3–4_H) is HDASH, which has been suggested as one of the characteristics of Δ5 desaturases and necessary for its function to convert DGLA to ARA [34]. Recent studies find that several Δ5 desaturases (GenBank accession #s: AAL82631, AAL13311, AAL92562, AAM09687, CAJ07076) do not contain the exact HDASH sequence. Due to the important role of Δ5 desaturases in LC-PUFA biosynthesis, a detailed understanding of the functional significance of the conserved HPGG and HDASH motifs may contribute to improvements in hosts biologically engineered to produce commercially valuable LC-PUFA.

We report the isolation of a Δ5 desaturase gene from *Euglena gracilis* (*EgΔ5D*). Expression of *EgΔ5D* in a genetically modified DGLA producing *Y. lipolytica* strain revealed that *EgΔ5D* had strong Δ5 desaturase activity. Functional dissection of HPGG and HDASH motifs demonstrated that neither the HPGG nor the HDASH motif is necessary in the exact form as encoded for enzyme activity of *EgΔ5D*. Various mutants, within HPGG or HDASH motif alone, or within both HPGG and HDASH motifs, are functionally equivalent or have higher Δ5 desaturase activity than the wild type *EgΔ5D*. Codon optimization of the N-terminal region of a double mutant *EgΔ5D*-*34G158G* and substitution of the arginine with serine at residue 347 effectively improved the enzyme’s substrate conversion.

## Materials and Methods

### Strains, Media and Growth Conditions

*E. gracilis* was kindly provided by Dr. Richard Triemer of Michigan State University (East Lansing, MI). *Euglena* growth (EG) media (per liter): 1 g sodium acetate, 1 g of beef extract, 2 g of Bacto^®^ tryptone and 2 g of Bacto^®^ yeast extract in 970 mL of water. After filter sterilizing, 30 mL of soil–water supernatant was aseptically added. A 1-mL aliquot of *E. gracilis* culture was transferred into 250 ml of EG Medium in a 500-mL glass bottle. The cultures were grown at 23 °C with a 16 h light, 8 h dark cycle for 2 weeks with no agitation.

**Fig. 2:**
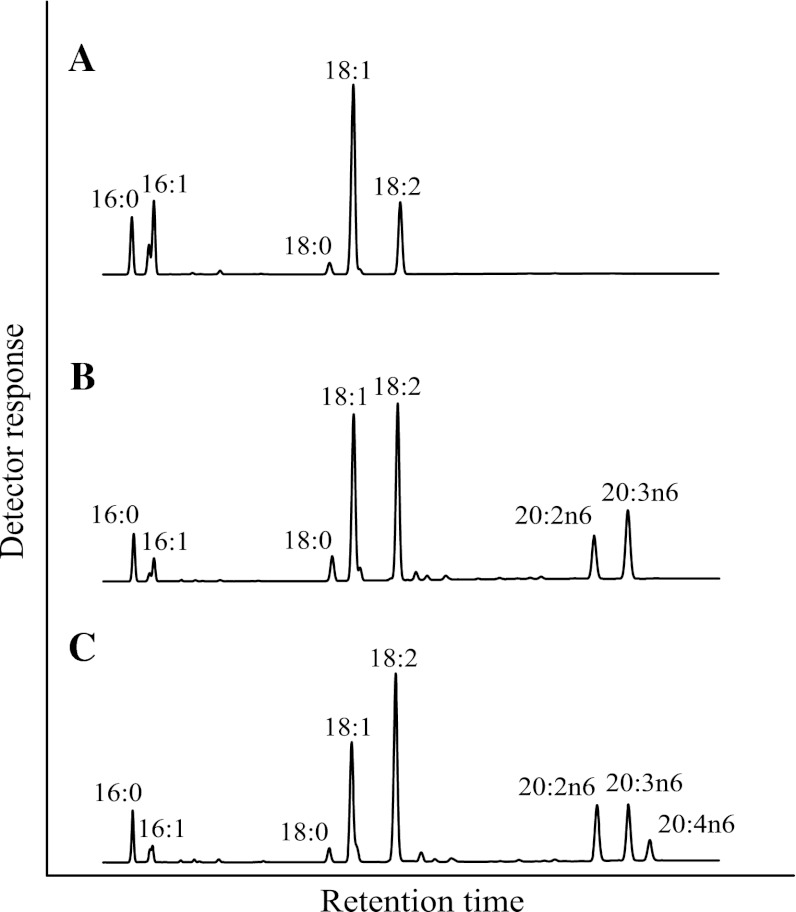
Chromatograms of the fatty acid profiles. Fatty acid profile of wild type *Y. lipolytica* strain ATCC#20362 (**a**). Fatty acid profile of Y4036U strain, producing about 8 % HGLA (**b**). Fatty acid profile of Y4036U strain transformed with pDMW367-M4, producing about 3.7 % ARA of total lipids (**c**)

*Y. lipolytica* strain Y2224 is a 5-fluoroorotic acid (FOA) resistant mutant of wild type strain American Type Culture Collection (ATCC, Rockville, MD) #20362 (Fig. 2a) with a mutation in the *URA3* gene (Genbank accession#: No. AJ306421). *Y. lipolytica* strain Y4036U (Zhu et al., unpublished data) is a genetically modified strain with a *Leu* ^−^ and *Ura* ^−^ phenotype, also originated from the wild type strain ATCC #20362. Strain Y4036U produced approximately 18 % DGLA of total fatty acids (Fig. 2b) and is composed of heterologous genes encoding Δ12 desaturase of *Fusarium moniliforme* [35]; C16/18 elongase of *M. alpina* [36]; Δ9 elongase of *E. gracilis* [37] and synthetic mutant of Δ8 desaturase [38] derived from *E. gracilis*. Minimal Media + Leucine (MMLeu), High Glucose Media (HGM) and YPD medium were used as required for *Y. lipolytica* strains and cultured at 30 °C. MMLeu (per liter): 20 g of glucose; 1.7 g yeast nitrogen base without amino acids or ammonium sulfate; 0.1 g proline; 0.1 g leucine; pH 6.1. HGM (per liter): 80 g glucose, 2.58 g KH_2_PO_4_, 5.36 g K_2_HPO_4_, pH 7.5. YPD medium (per liter): 10 g of yeast extract, 20 g of Bacto peptone, and 20 g of glucose. Agar plates were prepared by addition of 20 g/l agar to liquid media.

### General Techniques for Molecular Biology

Recombinant DNA techniques were used according to standard methods [39, 40]. Site-directed mutagenesis was performed according to the manufacturer’s protocol (QuikChange™, Stratagene; San Diego, CA). When PCR or site-directed mutagenesis was involved in the generation of mutants and/or cloning, DNA was sequenced to verify that no additional mutations were introduced.

Total RNA was extracted from the *E. gracilis* cells using the RNA STAT-60™ reagent (Amsbio LLC., Lake forest, CA). 85 µg of mRNA was purified from 1 mg of total RNA using the mRNA Purification Kit (Amersham Biosciences, Piscataway, NJ). Synthesis of cDNA from the *E. gracilis* mRNA was carried out using the adapter primer AP of 3′-RACE kit from Invitrogen (Carlsbad, CA) and the Smart IV oligonucleotide of BD-Clontech Creator™ Smart™ cDNA library kit (Mississauga, ON, Canada) as primers. The reverse transcription was performed with Superscript II reverse transcriptase of Invitrogen.

PCR reactions were carried out in a 50 µl total volume comprising: PCR buffer (containing 10 mM KCl, 10 mM (NH_4_)_2_SO_4_, 20 mM Tris–HCl (pH 8.75), 2 mM MgSO_4_, 0.1 % Triton X-100), 100 µg/mL BSA, 200 µM each deoxyribonucleotide triphosphate, 10 pmol of each primer, 10 ng cDNA of *E. gracilis* and 1 µl of Taq DNA polymerase (Epicentre Technologies, Madison, WI). The thermocycler conditions were set for 35 cycles at 95 °C for 1 min, 56 °C for 30 s and 72 °C for 1 min, followed by a final extension at 72 °C for 10 min. The DNA band with expected size was isolated from a 1 % agarose gel and cloned into pGEM-T easy vector (Promega, Madison, WI.).

Modified 5′ and 3′ RACE techniques were used to obtain the full length *EgΔ5D*. The cDNA product from *E. gracilis* mRNA was used as template, and all the primers used in the 5′ and 3′ RACE are listed in Supplemental Table S1. Specifically, a gene specific primer ODMWP480 and a generic primer CDSIII 5′ were used in the first round of 5′ RACE. The PCR amplifications were carried out in a 50 µl total volume, comprising: 25 µl of *LA Taq*™ pre-mix (TaKaRa Bio Inc., Otsu, Shiga, 520-2193, Japan), 10 pmol of each primer and 1 µl of Taq DNA polymerase (Epicentre Technologies, Madison, WI). The thermocycler conditions were the same as described above. One micro liter of this product was directly used in a second amplification, which differed from the first only in that the primers used, ODMWP479 and the generic primer DNR CDS 5′ were internal to the first set of primers. As no translation initiation codon was found in the product of the first round of 5′ RACE, the entire modified 5′ RACE protocol was repeated using gene specific primers YL791 and YL792 as primers instead of the primers used in the first round.

A variation of a 3′ RACE technique was used to isolate the C-terminal fragment of *EgΔ5D*. The combinations of primers ODMW469 and AUAP, and then YL470 and AUAP were used in the initial amplification and second round reaction, respectively. The PCR reactions were the same as those described for the 5′ RACE.

### *Yarrowia* Expression Vector and Transformation

*Yarrowia* expression vector pDMW367 contained autonomous replication sequence 18 [41] and a *URA3* gene (Genbank accession#: No. AJ306421) of *Y. lipolytica*. It also contains a *FBAIN::EgΔ5D:Pex20* chimeric gene. The FBAIN is a promoter derived from the fructose-bisphosphate aldolase gene (*FBA1*) of *Y. lipolytica* [42]. The *EgΔ5D* is the coding region of a wild type Δ5 desaturase of *E. gracilis*, in which the amino acid at position 347 is arginine. The Pex20 was a terminator sequence of *PEX20* gene (Genbank accession#: AF054613) of *Y. lipolytica*. Transformation of *Y. lipolytica* strain Y4036U was carried out as described by Chen et al. [43].

### Cultivation of *Y. lipolytica* Transformants and Fatty Acid Analysis by Gas Chromatography

The *Yarrowia* expression plasmid and its derivatives were used to transform strain Y4036U individually. Transformants from each transformation were streaked onto new MMLeu plates and kept in a 30 °C incubator for 2 days. Cells from streaked plates were cultivated in 24 well blocks with 3 mL MMLeu, and incubated for 2 days at 30 °C with shaking at 200 rpm. The cells were then collected by centrifugation and resuspended in 3 mL HGM. The cells were incubated another 5 days at 30 °C with shaking at 200 rpm.

Fatty acid methyl esters from 1 ml cell culture of *Y. lipolytica* or *E. gracilis* were prepared as described [44], except that the fatty acid methyl esters were extracted with 0.5 ml of heptane and separated by Agilent 7890A GC using hydrogen as carrier gas supplied by a hydrogen generator (Parker Hannifin, Cleveland, OH). The oven temperature was programmed from 200 to 240 °C at a rate of 25 °C/min. The proportion of each fatty acid was based on the integrated peak area of the corresponding fatty acid methyl ester as a percent relative to the sum of all integrated peaks as calculated by Agilent ChemStation Software.

## Results

### Isolation of Δ5 Desaturase Gene from *E. gracilis*

Fatty acid profile analyses showed that there were moderate amounts of EDA, DGLA, ARA, EPA, docosapentaenoic acid (DPA 22:5n-3) and DHA produced in *E. gracilis* (data not shown), confirming that there was a “Δ9 elongase/Δ8 desaturase” pathway in *E. gracilis* [16, 38, 45]. The genes encoding the Δ9 elongase, Δ8 desaturase [16, 38], Δ5 desaturase, Δ17 desaturase, C20/22 elongase and Δ4 desaturase [45] were responsible for the production of EDA, DGLA, ARA, EPA, DPA and DHA, respectively (Fig. 1).

**Fig. 3:**
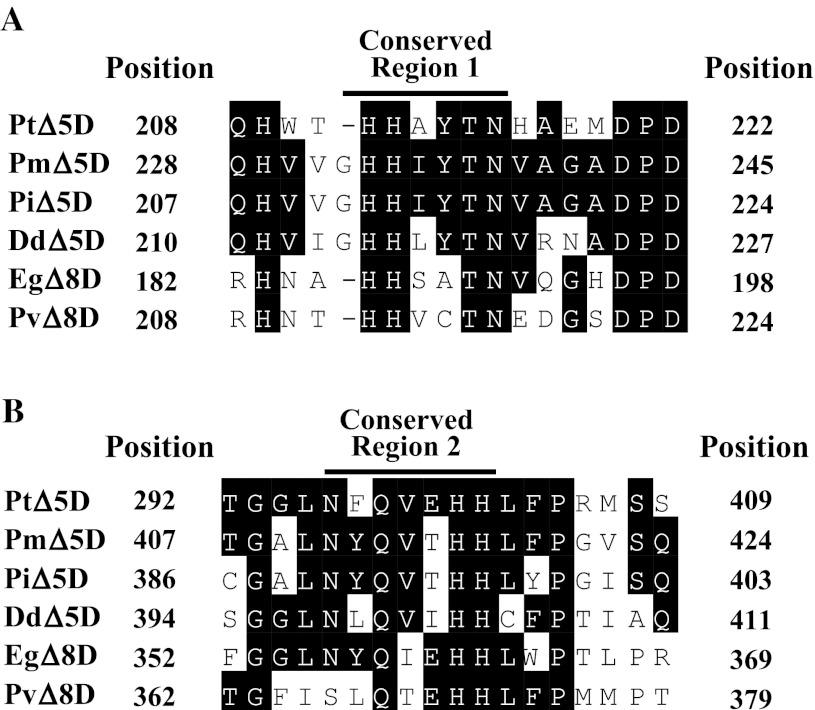
Alignment of the conserved regions among some Δ5 and Δ8 desaturases. The amino acid sequence alignment was performed with Clustal W analysis (MegAlign™ program of DNASTAR software). Identical residues are shaded in *black*. *PtΔ5D*, Δ5 desaturase from *P. tricornutum* (29, GenBank accession #: AAL92562); *PmΔ5D*, Δ5 desaturase from *P. megasperma* (GenBank accession #: CAD53323); *PiΔ5D*, Δ5 desaturase from *P. irregulare* (30, GenBack accession #: AAL13311); *DdΔ5D*, Δ5 desaturase from *D. discoideum* (GenBack accession #: XP_640311); *EgΔ8D*, Δ8 desaturase from *E. gracilis* (24, GenBack accession #: AAD45877) and *PvΔ8D*, Δ8 desaturase from *P. lutheri* [48]

After comparison of four Δ5 desaturase genes, *PiΔ5D* from *Pythium irregulare* [46], *PmΔ5D* from *Phytophthora megasperma* (Genbank accession #: CAD53323), *PtΔ5D* from *Phaeodactylum tricornutum* [47], and *DdΔ5D* from *Dictyostelium discoideum* (Genbank accession #: XP_640331) as well as two Δ8 desaturase genes, *EgΔ8D* from *E. gracilis* [16, 38] and *PlΔ8D* from *Pavlova lutheri* [48], two conserved regions, GHH(I/V)YTN and N(Y/F)Q(V/I)EHH (Fig. 3) were selected to design primers to amplify a portion of *EgΔ5D*. To reduce the degeneracy of the primers, four primers (Supplemental Table S1: 5-1A to 1D) were generated for conserved region 1 and four primers (Supplemental Table S1: 5-5AR to 5-5DR) for the anti-sense strand of conserved region 2. One DNA fragment amplified with primers 5-1B and 5-5DR was cloned into pGEM-T Easy vector to generate pT-F10-1. DNA sequence showed that a 590 bp insert of pT-F10-1 encoded an amino acid sequence with 38 % identity and 53 % similarity to the amino acid sequence of the Δ8-sphingolipid desaturase of *Thalassiosira pseudonana (TsΔ8D,* Genbank accession #: AAX14502), and 37 % identity and 52 % similarity with *PtΔ5D* [47]. These data suggested that the 590 bp DNA fragment might be a part of a desaturase gene of *E. gracilis.* This gene was designated as putative *EgΔ5D.*

**Fig. 4:**
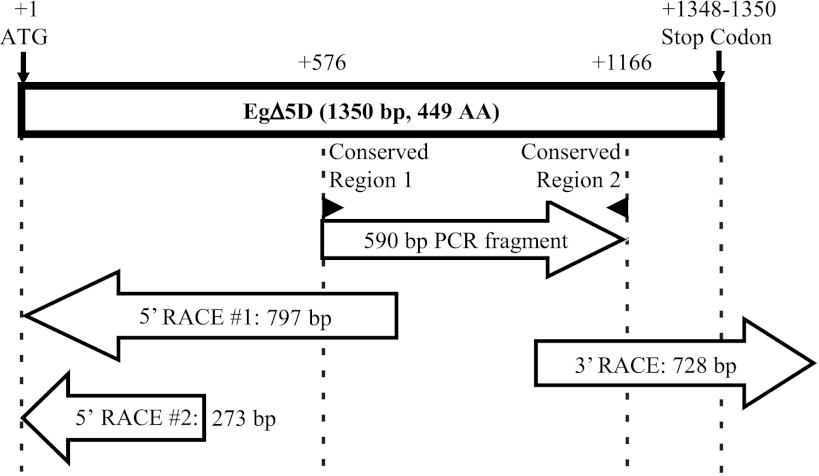
Graphical representation of the assembly of full length *EgΔ5D*. A 590 bp DNA fragment encoding a portion of *EgΔ5D* was isolated by PCR amplification with degenerate primers. The N-terminal part was isolated by two round of 5′ RACE, and the C-terminal portion was isolated by one round of 3′ RACE

5′ and 3′ RACE techniques were used to extend the 590 bp region of the putative *EgΔ5D* (Fig. 4). A 797 bp DNA fragment with no translation initiation codon was isolated by the first round of 5′ RACE. This 797 bp DNA fragment had a 238 bp overlap with the 5′ end of the 590 bp fragment of pT-F10-1 and provided 559 bp of 5′ upstream sequence. A 273 bp DNA fragment was generated by the second round 5′ RACE experiment. This 273 bp DNA fragment had 253 bp overlap with the 5′ part of the 797 bp DNA fragment and provided 20 bp of 5′ upstream sequence. Seventeen [17] bp of the 20 bp encoded the N-terminal portion of the putative *EgΔ5D*, including the translation initiation codon. A 464 bp DNA sequence was identified by one round of 3′ RACE. The first 184 bp of the 464 bp fragment encoded the C-terminal coding region, including the translation stop codon, of the putative *EgΔ5D*.

Assembly of the 5′ region, the original 590 bp fragment and the 3′ region resulted in a 1,633 bp contig, comprising the complete coding region with additional untranslated 5′ and 3′ ends (Fig. 4). The coding region of the putative *EgΔ5D* is 1,350 bp in length and encodes a peptide of 449 amino acids. BlastP searches using the full length putative *EgΔ5D* as the query sequence showed that it shares 39 % identity and 56 % similarity with *PtΔ5D* [47]; 37 % identity and 55 % similarity with *TsΔ8D* (Genbank accession #: AAX14502). Amino acid sequence alignment performed with the Clustal W analysis (MegAlign™ program of DNASTAR software) showed that *EgΔ5D* has <30 % identity with some represent Δ5 desaturases such as *IgΔ5D* from *I. galbana* (AEA72469); *MaΔ5D* from *M. alpina* [24, 25, 34]; *PiΔ5D* [46]; *OtΔ5D* from *O. tauri* (Genbank accession #: XP_003082424) and *TaΔ5D* from *T. aureum* [49]. Further analyses showed that *EgΔ5D* has only 20 % and 25.5 % identity with *EgΔ8D* [16] and *EgΔ4D* [45] desaturases of *E. gracilis*, respectively. It was found that the PCR products for the full length coding region of putative *EgΔ5D* had two versions, both having identical nucleotide sequence except at base pair positions 1,039 and 1,041. This disagreement resulted in a codon change from CGA to AGC. As such, one PCR product indicated arginine at position 347, whereas the second indicated serine. It was hypothesized that this discrepancy was raised at the stage of PCR amplification or during cDNA generation.

### Determination of Δ5 Desaturase Activity and Topology Model of *EgΔ5D*

To study the function of the putative *EgΔ5D*, plasmid pDMW367 was generated to express the *EgΔ5D* coding region under the control of the strong constitutive FBAIN promoter [42] from *Y. lipolytica*. The four restriction sites (i.e., *Bgl*II, *EcoR*I, *Hin*dIII and *Nco*I) inside the coding region of *EgΔ5D* in pDMW367 were removed by site-directed mutagenesis to generate pDMW367-M4 (Supplemental Fig. S1). The amino acid sequence of *EgΔ5D* is identical in pDMW367 and pDMW367-M4 constructs, and the amino acid at position 347 is an arginine.

The pDMW367 and pDMW367-M4 constructs were used to transform DGLA producing strain Y4036U. There was no ARA produced in the parent strain Y4036U (Fig. 2b), while there was about 3.7 % ARA and 11.5 % DGLA produced in transformants of Y4036U with either the pDMW367 or pDMW367-M4 construct (Fig. 2c). These results demonstrated that *EgΔ5D* indeed encodes a Δ5 desaturase. *EgΔ5D* could convert DGLA to ARA with a conversion of about 24.3 %. The conversion of DGLA to ARA was calculated according to the formula: ([ARA product]/[DGLA substrate + ARA product]). The pDMW367 and pDMW367-M4 constructs were also used to transform strain Y2224, a FOA resistant mutant of wild type strain ATCC#20362. There was no GLA produced in the transformants with either the pDMW367 or pDMW367-M4 construct (data not shown). These results demonstrated that *EgΔ5D* does not have Δ6 desaturase activity; it is not a bi-functional enzyme.

**Fig. 5:**
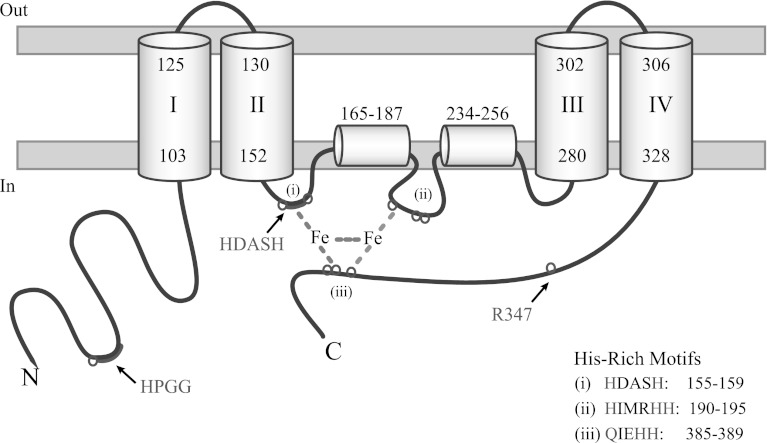
Topology model of *EgΔ5D*. *EgΔ5D* is a membrane diiron protein with three His-rich motifs. The HX_(3, 4)_H motif, HDASH, is located from residues 155 to 159; the HX_(2, 3)_HH motif, HIMRHH, located from residues 190 to 195, and the (H/Q)X_(2, 3)_HH motif, QIEHH, located from residues 385 to 389. *EgΔ5D* has four trans-membrane domains (I–IV) and two hydrophobic stretches (residues from 165 to 187, and 234 to 256). Its N-terminal cytochrome *b*_5_ domain and C-terminal region are located in the cytoplasm

Like other fatty acid desaturases, *EgΔ5D* is also a membrane-bound enzyme and belongs to a super-family of membrane di-iron proteins with three His-rich motifs: HX_(3, 4)_H, HX_(2, 3)_HH and (H/Q)X_(2, 3)_HH. These His residues have been predicted to be located in the cytoplasmic face of the membrane and have been shown to be very important for enzyme activity [33]. Within *EgΔ5D*, these 3 His-rich motifs are the HDASH motif located from residue 155 to 159, the HIMRHH motif located from residue 190 to 195, and the QIEHH motif located from residue 385 to 389. The third His-rich motif contains a glutamine substitution that is common to other front-end desaturases. Based on transmembrane domain analysis (TMHMM Server v. 2.0, Center for Biological Sequence Analysis, BioCentrum-DTU, Technical University of Denmark, DK-2800 Lyngby, Denmark) and the location of the His-rich motifs, a topology model of *EgD5D* was developed (Fig. 5). The model shows that the N-terminal cytochrome *b*_5_ domain is located in the cytosol. The topology model also predicts that *EgΔ5D* has a total of four transmembrane regions (amino acid residues 103–125, 130–152, 280–302 and 306–328) and two hydrophobic regions (amino acid residues 165–187, and 234–256). These hydrophobic segments are not membrane-spanning, and may represent hydrophobic patches located closed to the di-iron active site. Because the substrates for the desaturase is highly hydrophobic, they will likely partition into the lipid bilayer. Therefore we purport that the di-iron active site assembled from these His-clusters may occur at or very near the membrane surface.

### The HPGG Motif is Important, but not Necessary for Δ5 Desaturase Activity of *EgΔ5D*

It has been suggested that the highly conserved HPGG motif plays a crucial role in heme group assembly, protein folding and stabilization in cytochrome *b*_5_ proteins, with the histidine residue functioning as an axial heme ligand where a peptide chain reversal occurs [50]. Previous studies have demonstrated that the heme-binding HPGG motif, and in particular, the histidine residue, is essential for enzyme activity of desaturases with cytochrome *b*_5_ domain [51–53]. Although sequence divergence in the vicinity of the HPGG motif is normal, the HPGG motif itself has been conserved throughout the evolution of all the Δ5 desaturase genes [54]. Thus it was claimed to be a characteristic of Δ5 desaturases and necessary for its function to convert DGLA to ARA [34].

To assess the functional significance of the HPGG motif (position 33–36) of *EgΔ5D*, we first elected the proline residue at position 34 (P34) as a target for amino acid substitution. Single amino acid mutations were carried out to generate all 19 amino acid substitution mutants (HxGG). Table 1 shows that the P34 residue could be substituted with several different amino acids without significantly impacting the Δ5 desaturase activity of *EgΔ5D*. The *EgΔ5D*-*34A, EgΔ5D*-*34C, EgΔ5D*-*34K* or *EgΔ5D*-*34W* mutants exhibited >90 % of the wild type *EgΔ5D* activity. The *EgΔ5D*-*34G* mutant was functionally equivalent to the wild type *EgΔ5D*.

**Table 1:** Δ5 Desaturase activity of *EgΔ5D* with HxGG mutations

Gene name	Sequence of HPGG motif	Δ5 Conversion (%)	% of wild type EgΔ5D
*EgΔ5D*	HPGG	24.2	100
*EgΔ5D-34A*	HaGG	22.7	93.5
*EgΔ5D-34C*	HcGG	22.6	93.2
*EgΔ5D-34D*	HdGG	12.5	51.6
EgΔ5D-34E	HeGG	14.7	60.5
*EgΔ5D-34F*	HfGG	17.9	73.9
*EgΔ5D-34G*	HgGG	23.8	98.2
*EgΔ5D-34H*	HhGG	21.3	87.8
*EgΔ5D-34I*	HiGG	18.3	75.4
EgΔ5D-34K	HkGG	22.7	93.8
*EgΔ5D-34L*	HlGG	17.0	70.0
*EgΔ5D-34M*	HmGG	19.0	78.4
*EgΔ5D-34N*	HnGG	19.8	81.5
*EgΔ5D-34Q*	HqGG	19.8	81.7
*EgΔ5D-34R*	HrGG	19.4	79.9
*EgΔ5D-34S*	HsGG	20.4	84.1
*EgΔ5D-34T*	HtGG	19.6	80.7
*EgΔ5D-34V*	HvGG	20.2	83.4
*EgΔ5D-34W*	HwGG	22.2	91.7
*EgΔ5D-34Y*	HyGG	17.7	73.1

Average of 6 samples for each construct containing different mutations

Next, we studied the significance of the second glycine residue at position 36 (G36) within the HPGG motif of *EgΔ5D* using the same approach as that for P34. Table 2 shows that the G36 residue within the HPGG motif could be substituted with several different amino acids without significantly impacting the Δ5 desaturase activity of *EgΔ5D*. The *EgΔ5D*-*36S* or *EgΔ5D*-*36D* mutant had about 100.8 or 99.2 % of Δ5 desaturase activity when compared to *EgΔ5D*, respectively.

**Table 2:** Δ5 Desaturase activity of *EgΔ5D* with HPGx mutations

Gene name	Sequence of HPGG motif	Δ5 Conversion (%)	% of wild type EgΔ5D
*EgΔ5D*	HPGG	24.2	100
*EgΔ5D-36A*	HPGa	18.3	75.6
*EgΔ5D-36C*	HPGc	6.5	26.8
*EgΔ5D-36D*	HPGd	24.0	99.2
*EgΔ5D-36E*	HPGe	21.1	86.9
*EgΔ5D-36F*	HPGf	3.3	13.4
*EgΔ5D-36H*	HPGh	18.1	74.8
*EgΔ5D-36I*	HPGi	1.5	6.4
*EgΔ5D-36K*	HPGk	19.0	78.3
*EgΔ5D-36L*	HPGl	9.1	37.7
*EgΔ5D-36M*	HPGm	13.4	55.2
*EgΔ5D-36N*	HPGn	19.5	80.3
*EgΔ5D-36P*	HPGp	18.0	74.1
*EgΔ5D-36Q*	HPGq	19.9	82.1
*EgΔ5D-36R*	HPGr	15.5	63.8
*EgΔ5D-36S*	HPGs	24.4	100.8
*EgΔ5D-36T*	HPGt	22.8	93.9
*EgΔ5D-36V*	HPGv	1.9	8.0
*EgΔ5D-36W*	HPGw	15.1	62.2
*EgΔ5D-36Y*	HPGy	11.2	46.3

Average of 6 samples for each construct containing different mutations

The above functional studies at the P34 and G36 positions within the HPGG motif of *EgΔ5D* demonstrated that the HPGG motif could be changed without impacting the Δ5 desaturase activity. Specifically, the *EgΔ5D*-*34G*, *Eg*Δ*5D*-*36S* and *EgΔ5D*-*36D* mutants were functionally equivalent to the wild type *EgΔ5D*. Comparison of the ARA in unesterified fatty acid (FFA), phospholipid (PL) and neutral lipid (NL) pools of *Yarrowia* transformants with *EgΔ5D* and *EgΔ5D*-*34G* showed that the P34G mutation did not affect the ARA distribution in these pools (Supplemental Fig. S2).

### Improvement of Δ5 Desaturase Activity of *EgΔ5D* by Amino Acid Substitution Within the HDASH Motif

The HDASH motif was also claimed as one of the characteristics of Δ5 desaturases and necessary for its function to convert DGLA to ARA [34]. To test the hypothesis that the exact sequence of the HDASH motif (position 155–159) of *EgΔ5D* was required, we first selected the alanine residue at position 157 (A157) as a target. The Δ5 desaturase activity attributed to each mutation at A157 is summarized in Table 3. The data showed that almost all mutations at A157 greatly reduced the Δ5 desaturase activity of *EgΔ5D*. However, the *EgΔ5D*-*157G* and *EgΔ5D*-*157S* mutants retained about 96 and 94 % activity of wild type *EgΔ5D*, respectively.

**Table 3:** Δ5 Desaturase activity of *EgΔ5D* with HDxSH mutations

Gene name	Sequence of HDASH motif	Δ5 Conversion (%)	% of wild type EgΔ5D
*EgΔ5D*	HDASH	24.8	100
*EgΔ5D-157C*	HDcSH	10.7	43.1
*EgΔ5D-157D*	HDdSH	1.0	4.0
*EgΔ5D-157E*	HDeSH	0.9	3.6
*EgΔ5D-157F*	HDfSH	1.0	4.0
*EgΔ5D-157G*	HDgSH	23.8	96
*EgΔ5D-157H*	HDhSH	1.0	4.0
*EgΔ5D-157I*	HDiSH	0.9	3.6
*EgΔ5D-157K*	HDkSH	1.0	4.0
*EgΔ5D-157L*	HDlSH	1.1	4.4
*EgΔ5D-157M*	HDmSH	1.0	4.0
*EgΔ5D-157N*	HDnSH	1.1	4.4
*EgΔ5D-157P*	HDpSH	2.3	9.3
*EgΔ5D-157Q*	HDqSH	0.6	2.4
*EgΔ5D-157R*	HDrSH	0.8	3.2
*EgΔ5D-157S*	HDsSH	23.3	94
*EgΔ5D-157T*	HDtSH	1.0	4.0
*EgΔ5D-157V*	HDvSH	0.3	1.2
*EgΔ5D-157W*	HDwSH	0.9	3.6
*EgΔ5D-157Y*	HDySH	0.7	2.8

Average of 3 samples for each construct containing different mutations

We also studied the significance of the serine residue at position 158 (S158) within the HDASH motif of *EgΔ5D*. Table 4 shows that the S158 could be substituted with either an alanine or a glycine without substantially impacting the ∆5 desaturase activity of *EgΔ5D*. Specifically, the *EgΔ5D*-*158A* mutant had about 100.9 % activity of wild type *EgΔ5D*, while the *EgΔ5D*-*158G* mutation had about 107.7 % activity of wild type *EgΔ5D*. These data demonstrated that the HDASH motif could be changed without significantly reducing the Δ5 desaturase activity. In fact, the enzyme activity of *EgΔ5D* could be improved by substitution of the S158 within the HDASH motif with a glycine residue.

**Table 4:** Δ5 Desaturase activity of *EgΔ5D* with HDAxH mutations

Gene name	Sequence of HDASH motif	Δ5 Conversion (%)	% of wild type EgΔ5D
*EgΔ5D*	HDASH	23.3	100
*EgΔ5D-158A*	HDAaH	23.5	100.9
*EgΔ5D-158C*	HDAcH	17.9	76.8
*EgΔ5D-158D*	HDAdH	2.8	12.0
*EgΔ5D-158E*	HDAeH	1.9	8.2
*EgΔ5D-158F*	HDAfH	1	4.3
*EgΔ5D-158G*	HDAgH	25.1	107.7
*EgΔ5D-158H*	HDAhH	1.6	6.9
*EgΔ5D-158I*	HDAiH	1.1	4.7
*EgΔ5D-158K*	HDAkH	1	4.3
*EgΔ5D-158L*	HDAlH	1.1	4.7
*EgΔ5D-158M*	HDAmH	2.3	9.9
*EgΔ5D-158N*	HDAnH	16.5	70.8
*EgΔ5D-158P*	HDApH	1.2	5.2
*EgΔ5D-158Q*	HDAqH	10.4	44.6
*EgΔ5D-158R*	HDArH	10.0	42.9
*EgΔ5D-158T*	HDAtH	9.6	41.2
*EgΔ5D-158V*	HDAvH	1.5	6.4
*EgΔ5D-158W*	HDAwH	9.3	40.0
*EgΔ5D-158Y*	HDAyH	1.1	4.7

Average of 3 samples for each construct containing different mutations

### Both HPGG and HDASH Motifs were not Necessary in the Exact Form as Encoded for Δ5 Desaturase Activity of *EgΔ5D*

In order to study whether *EgΔ5D* could maintain strong Δ5 desaturase activity with mutations in both HPGG and HDASH motifs, we generated a series of double mutants. The results (Table 5) demonstrate that ∆5 desaturases could be constructed having variant HPGG and HDASH motifs that retain at least 64 % of ∆5 desaturase activity when compared to the wild type.

**Table 5:** Δ5 Desaturase activity of *EgΔ5D* with mutants simultaneously comprising mutations within HPGG and HDASH motifs

Gene name	Sequence of HPGG motif	Sequence of HDASH motif	Δ5 Conversion (%)	% of wild type EgΔ5D
*EgΔ5D*	HPGG	HDASH	27.5	100
*EgΔ5D-*34G157G	HgGG	HDgSH	22.9	83
*EgΔ5D-34G158A*	HgGG	HDAaH	24.3	88
*EgΔ5D-34G158G*	HgGG	HDAgH	26.8	97
*EgΔ5D-34H158A*	HhGG	HDAaH	18.7	68
*EgΔ5D-34H158A*	HhGG	HDAgH	22	80
*EgΔ5D-36S158A*	HPGs	HDAaH	17.5	64
*EgΔ5D-36S158G*	HPGs	HDAgH	18.9	69

Average of 6 samples for each construct containing different mutations

The proline residue within the HPGG motif can be substituted with glycine with simultaneous substitution of either (1) the alanine residue within the HDASH motif for glycine or (2) the serine residue within the HDASH motif for alanine or glycine. The proline residue within the HPGG motif can also be substituted with histidine with simultaneous substitution of the serine residue within the HDASH motif for either alanine or glycine. And, the second glycine residue within the HPGG motif can be substituted with serine with simultaneous substitution of the serine residue within the HDASH motif for either alanine or glycine. Specifically, the *EgΔ5D*-*34G/157G, EgΔ5D*-*34G/158A* and *EgΔ5D*-*34H/158G* double mutants had more than 80 % of the Δ5 desaturase activity of *EgΔ5D*, while *EgΔ5D*-*34G/158G* had about 97 % Δ5 desaturase activity of *EgΔ5D*. Further analyses showed that the ARA distribution in FFA, PL and NL pools of *Yarrowia* transformants with *EgΔ5D*-*34G/158G* was similar to *Yarrowia* transformants with *EgΔ5D*-*34G* or *EgΔ5D* (Supplemental Fig. S2), suggesting that the simultaneous substitutions (P34G and S158G) within HPGG and HDASH motifs did not change desaturase substrate specificity.

### Increased Substrate Conversion of *EgΔ5D*-*34G/158G* with Double Mutations in HPGG and HDASH Motifs

**Fig. 6:**
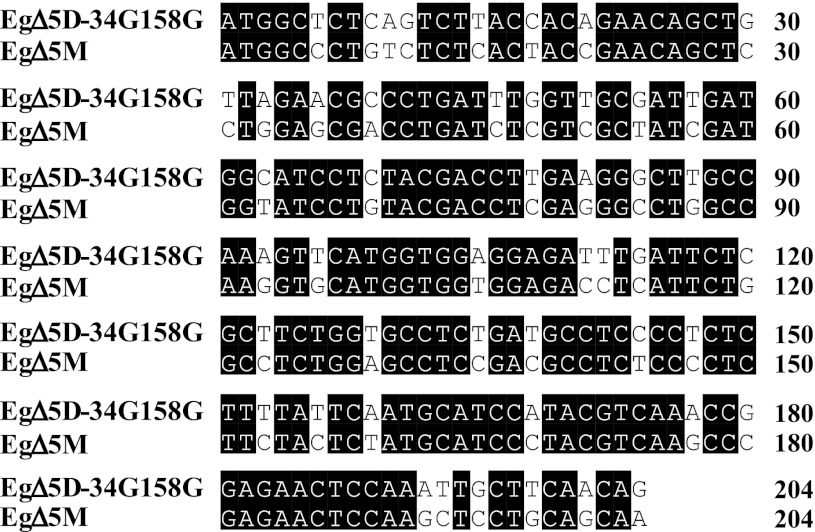
Comparison of the 5′ portion (204 bp) of *EgΔ5D*-*34G158G* and its codon optimized version, *EgΔ5M*. The DNA sequence alignment was performed with Clustal W analysis (MegAlign™ program of DNASTAR software). Identical residues are shaded in *black*

In order to increase the substrate conversion of *EgΔ5D*-*34G/158G*, we optimized the codon usage of the N-terminal portion of the gene for expression in *Y. lipolytica.* The codon-optimized *EgΔ5D*-*34G/158G*, designated as “*EgΔ5M*”, had 48 bp changed in the first 204 bp of the coding region (23.5 %; Fig. 6), which resulted in optimization of 43 codons of the first 68 amino acids within the N-terminus of the protein (63.2 %). The amino acid sequence encoded by the codon-optimized *EgΔ5M* was identical to that of the *EgΔ5D*-*34G/158G*. *EgΔ5M* was used to replace the *EgΔ5D* of pDMW367-M4 to generate pDMW367-5M, containing a *FBAIN::EgΔ5M::Pex20* chimeric gene.

We then studied the importance of the arginine or serine at position 347 that were found in the original clones of *EgΔ5D*. Based on *EgΔ5M*, the CGA codon for arginine at position 347 was changed to AGC codon to encode for serine, which was designated as *EgΔ5M1*. The synthetic *EgΔ5M1* was used to replace the *EgΔ5D* of pDMW367-M4 to generate pDMW367-5M1, containing an *FBAIN::EgΔ5M1::Pex20* chimeric gene.

The Δ5 desaturase activity of *EgΔ5D, EgΔ5M* and *EgΔ5M1* is summarized in Table 6. GC analyses determined that there were about 3.6 % ARA and 10.8 % DGLA, 4.0 % ARA and 11.2 % DGLA, and 4.1 % ARA and 10.8 % DGLA of total fatty acids produced in the *Yarrowia* transformants with pDMW367-M4, pDMW367-5M, and pDMW367-5M1, respectively. It showed that the wild-type *EgΔ5D* converted about 24.8 % of DGLA to ARA; *EgΔ5M* converted 26.7 % of DGLA to ARA; and, *EgΔ5M1* converted 27.6 % of DGLA to ARA. The fatty acid profile of *Yarrowia* transformants with *EgΔ5M1* was almost identical to the profile of *Yarrowia* transformants with pDMW367-M4 as shown in Fig. 2c, except that more ARA was produced. These data demonstrated that the codon optimization of *EgΔ5D* improved its substrate conversion efficiency. Further, the amino acid at position 347 did affect the ∆5 desaturase activity of *EgΔ5D*, with a serine residue preferred over an arginine residue.

## Discussion

*Y. lipolytica* has an established history of robust fermentation performance at commercial scale for processes including the production of food-grade citric acid for human consumption and single-cell protein for animal feeds [55]. Recently, *Y. lipolytica* has been used as a host for production of lipid-based compounds [14, 56, 57]. Some *Y. lipolytica* strains are oleaginous organisms that are able to accumulate up to 40 % dry cell weight as oil when starved for nitrogen in the presence of excess glucose as carbon source. However, LNA is the only PUFA that *Y. lipolytica* can synthesize de novo (Fig. 2a). Therefore, it is necessary to isolate genes encoding enzymes for every step of the “desaturation and elongation” pathways (Fig. 1) before genetically engineering *Y. lipolytica* to produce ARA, EPA and DHA oil. Δ5 desaturase is the enzyme responsible for the conversion of DGLA to ARA, and ETA to EPA. Although several Δ5 desaturase genes have been isolated from various organisms [26], more effective enzymes may help to improve the production of commercially important LC-PUFA.

Previous studies have indicated that *Euglena* was able to synthesize ARA, EPA and DHA through the “Δ9 elongase/Δ8 desaturase” pathway [16, 38, 45]. In this report, the gene encoding a Δ5 desaturase from *E. gracilis* was isolated and characterized. Our results indicated two nucleotide sequences with a difference of two base pairs that would result in either arginine or serine at position 347. This discrepancy was most likely generated from PCR amplification or during cDNA generation. BlastP searches showed that the amino acid sequence of *EgΔ5D* shares <40 % identity with any Δ5 desaturase found in Genbank; *PtΔ5D* [47] was the most similar one (about 39 %), suggesting that the primary structure of *EgΔD5* is quite different from those Δ5 desaturase genes previously isolated. Amino acid sequence alignment also shows that *EgΔ5D* has about 20 % identity with *EgΔ8D* [16, 38] and about 25.5 % identity with *EgΔ4D* [45]. These data suggest that *EgΔ5D* is evolutionary closer to Δ5 desaturase than the Δ4 or Δ8 desaturases. Functional analyses of *EgΔ5D* in *Y. lipolytica* strains Y4036U and Y2224 revealed that it has strong Δ5 desaturase activity, with more than 24 % substrate conversion of DGLA to ARA, and it is not a Δ5/Δ6 bifunctional enzyme.

The HPGG motif is expected to be on the cytochrome *b*_5_ surface, in contact with the heme through van der Waals interactions [27]. The conserved HPGG motif was thought to be essential in maintaining cytochrome *b*_5_ electron transfer function, with the histidine serving as a heme axial ligand [50]. Substitution of the histidine residue of the HPGG motif with alanine in the Δ6 desaturase cytochrome *b*_5_ domain of starflower, rat and algae abolished Δ6 desaturase activity [51–53]. It is expected that H33 of *EgΔ5D* should also be essential for its function.

The HPGG motif itself has a unique structure. The proline residue of the HPGG stretch is located in a turn between two consecutive helices (Fig. 5) and was thought to be important in protein folding and in maintaining cytochrome *b*_5_ protein stability [32]. The three-carbon side chain of proline is bonded to both the nitrogen and the carbon of the peptide backbone to form a five-member ring that greatly restricted its conformational freedom. The nonpolar characteristic of this ring structure may create a hydrophobic spot within the HPGG motif. On the other hand, the glycine possesses the smallest amino acid side chain, hydrogen, which can allow for greater flexibility in local structure. It is likely that the combination of proline and glycine residues within the cytochrome *b*_5_ HPGG motif is an important factor affecting both the structural position of the hydrophobic heme pocket and the appropriate orientation of the heme group within the heme pocket relative to the desaturase catalytic site. Surprisingly, our results indicate that the proline residue of the HPGG motif is not essential for electron transfer from the heme group of the cytochrome *b*_5_ domain to the catalytic diiron cluster of *EgΔ5D.* Most substitution mutants at P34 displayed at least 70 % of the wild type *EgΔ5D* activity. The *EgΔ5D*-*34G* (HgGG) mutant had greater than 98 % of the wild type *EgΔ5D* activity, demonstrating that the proline residue of HPGG motif is not required for the enzyme activity of *EgΔ5D* (Table 1).

It is noteworthy that aspartate substitutions in *EgΔ5D*-*34D* and *EgΔ5D*-*36D* exhibit different effects on desaturase activity. Compared to free cytochrome *b*_5_, while there are several conserved acidic amino acids, there is a characteristic reduction in the number of aspartate and glutamate residues in the vicinity of the HPGG motif of cytochrome *b*_5_ domain of desaturases. This reduced number of acidic residues around the heme pocket is thought to contribute to stabilizing nonpolar intermolecular interactions between the cytochrome *b*_5_ and desaturase domains [27, 54]. Substitutions involving aspartate or glutamate residues in the HPGG motif may affect the interface geometry of electron donor/acceptor docking that is exhibited in desaturase activity due to altered electron transfer. We also found that substitutions for G36 of *EgΔ5D* resulted in mutants with strong Δ5 desaturase activity. The most functional mutants were the small, slightly polar serine replacement, *EgΔ5D*-*36S*, and the acidic substitution with aspartate, *EgΔ5D*-*36D*. The activities of these two mutants are about the same as the wild type *EgΔ5D* (Table 2).

The amino acid sequence of the first His-rich motif, HX_(3, 4)_H, of *EgΔ5D* is HDASH located from residues 155 to 159. The HDASH motif has been suggested as one of the characteristics of Δ5 desaturases and necessary for its function to convert DGLA to ARA in any transformed organisms [34]. Sequence analyses showed that there are natural variants of the HDASH motif in Δ5 desaturases, for example, *PiΔ5D* [46] has the sequence of HDsSH, the Δ5 desaturase from *Thraustochytrium sp.* ATCC 21685 (GenBank accession #: AAM09687) has the sequence of HemgH, the Δ5 desaturase from *Leishmania major strain Friedlin* (GenBank accession #: CAJ07076) has the sequence of HeAgH, the Δ5 desaturase from Atlantic salmon (GenBank accession #: AAL82631) has the sequence of HDygH, and *PtΔ5D* [47] has the sequence of HDAnH in the corresponding location. This suggests that the HDASH motif is not an invariant characteristic of Δ5 desaturases, and may be not required for Δ5 desaturase activity. We suggest that the two His residues of HDASH motif participate in the coordination of the diiron center (Fig. 5), but the other three residues (DAS) residues between the two His residues can be modified.

Systemic substitution studies (Tables 3, 4) at positions A157 and S158 within the HDASH motif of *EgΔ5D* demonstrated that these two residues could be replaced, and the mutants retained good Δ5 desaturase activity. The *EgΔ5D*-*157G* and *EgΔ5D*-*157S* mutants had about 96 and 94 % of the wild type *EgΔ5D* activity, respectively. Since *PiΔ5D* has an HDsSH motif [46], it is not surprising that *EgΔ5D*-*157S* with sequence HDsSH functioned well in *Yarrowia*. We also found that S158 could be substituted with either alanine or glycine. The alanine, glycine and serine are interchangeable within the HDASH motif of *EgΔ5S;* furthermore, the enzyme activity of *EgΔ5D* could be improved with a motif of HDAgH instead of HDASH (Table 4).

To determine whether at least one motif, HPGG or HDASH, is required for the enzyme activity of *EgΔ5D*, a series of mutants with mutations in both the HPGG and HDASH motifs was generated (Table 5). Some double mutants such as *EgΔ5D*-*34G/157G, EgΔ5D*-*34G/158A* and *EgΔ5D*-*34H/158G* had more than 80 % of wild type *EgΔ5D* activity, while *EgΔ5D*-*34G/158G* had almost the same activity as wild type *EgΔ5D*. Therefore, neither the HPGG nor the HDASH motif is necessary in the exact form as encoded for the activity of *EgΔ5D*.

Distribution analyses (Supplemental Fig. S2) of ARA in FFA, PL and NL pools of *Yarrowia* transformants with *EgΔ5D* shows that more ARA loaded in PL pool than that in FFA pool, suggesting that *EgΔ5D* is also an acyl-lipid desaturase, just like other front-end desaturases from lower plants, fungi and algae [26]. ARA distribution comparison of *Yarrowia* transformants with *EgΔ5D*-*34G and EgΔ5D*-*34G/158G* with wild type *EgΔ5D* shows that either single mutation within HPGG motif (P34G), or simultaneous mutations within HPGG (P34G) and HDASH (S158G) motifs does not change its fatty acid distribution pattern (Supplemental Fig. S2), and therefore these changes in *EgΔ5D* do not affect its substrate specificity.

An effective Δ5 desaturase is required for efficient conversion of DGLA to ARA or ETA to EPA (Fig. 1) in engineered *Y. lipolytica* or other organisms to produce commercially valuable LC-PUFA. We employed two approaches to improve the enzyme activity of the double mutant *EgΔ5D*-*34G/158G*. The optimization (Fig. 6) of the 43 codons of the 68 amino acids within the N-terminal portion of *EgΔ5D*-*34G/158G, EgΔ5M*, improved substrate conversion (Table 6). This improvement may relate to the rate of translation. Recent reports suggest that sequence at the beginning of a gene can influence translation, and the mRNA structure at the 5′ end of an mRNA can affect protein levels [58, 59]. Next, we substituted the arginine residue at position 347 with the serine which was identified in our original PCR products. Surprisingly, this R347S substitution in codon optimized *EgΔ5M1* further improved substrate conversion (Table 6). These data suggest that some un-conserved amino acids among different Δ5 desaturases may be good targets for protein evolution to generate improved enzymes. At this stage, the improved *EgΔ5D,* both *EgΔ5M* and *EgΔ5M1* should enable us to engineer *Yarrowia* and other organisms to produce high levels of ARA, EPA and DHA.

**Table 6:** Δ5 Desaturase activity of *EgΔ5D* and codon optimized *EgΔ5M* and *EgΔ5M1*

Gene name	Sequence of HPGG motif	Sequence of HDASH motif	Sequence at residue 347	Δ5 Conversion (%)
*EgΔ5D*	HPGG	HDASH	R	24.8
*EgΔ5M*	HgGG	HDAgH	R	26.5
*EgΔ5M1*	HgGG	HDAgH	S	27.6

Average of 6 samples for each construct containing different mutations

In conclusion, our studies suggest that the exact sequences of the HPGG and HDASH motifs are not necessary for the function of *EgΔ5D*. Several amino acids within these two motifs can be changed individually, or simultaneously, without significantly reducing the enzyme activity or altering its substrate specificity. In some cases such as *EgΔ5D*-*36D*, *EgΔ5D*-*36S*, and *EgΔ5D*-*158G* mutants, the Δ5 desaturase activity can be improved. In order to fully understand the function of *EgΔ5D*, the roles of the HX_(2, 3)_HH and (H/Q)X_(2, 3)_HH motifs as well as the un-conserved amino acids need to be studied in the future.

## Electronic supplementary material

Below is the link to the electronic supplementary material.

Supplementary material 1 (DOC 103 kb)

## Abbreviations

ALA: Alpha-linolenic acid (ALA 18:3n-3)
ARA: Arachidonic acid (C20:4n-6)
ATCC: American Type Culture Collection (Rockville, MD)
DAG: Diacylglycerol
DdΔ5D: Δ5 Desaturase of *Dictyostelium discoideum*
DGLA: Dihomo-gamma-linoleic acid (C20:3n-6)
DHA: Docosahexaenoic acid (C22:6n-3)
DPA: Docosapentaenoic acid (22:5n-3)
EgΔ4D: Δ4 Desaturase of *Euglena gracilis*
EgΔ5D: Δ5 Desaturase of *Euglena gracilis*
EgΔ8D: Δ8 Desaturase of *Euglena gracilis*
EG Media: *Euglena* growth media
FFA: Unesterified fatty acids
FOA: 5-Fluoro-orotic acid FOA
EPA: Eicosapentaenoic acid (C20:5n-3)
ETA: Eicosatetraenoic acid (C20:4n-3)
GC: Gas chromatography
HGM: High glucose media
His-rich motif: Histidine-rich motif
IgΔ5D: Δ5 Desaturase of *Isochrysis galbana*
LC-PUFA: Long chain polyunsaturated fatty acids
LNA: Linoleic acid (18:2n-6)
MaΔ5D: Δ5 Desaturase of *Mortierella alpina*
MMLeu: Minimal media + leucine
NL: Neutral lipids
OtΔ5D: Δ5 Desaturase of *Ostreococcus tauri*
PCR: Polymerase chain reaction
PiΔ5D: Δ5 Desaturase of *Pythium irregulare*
PL: Phospholipids
PlΔ8D: Δ8 Desaturase of *Pavlova lutheri*
PmΔ5D: Δ5 Desaturase of *Phytophthora megasperma*
PtΔ5D: Δ5 Desaturase of *Phaeodactylum tricornutum*
PUFA: Polyunsaturated fatty acids
RACE: Rapid amplification of cDNA ends
TAG: Triacylglycerol
TaΔ5D: Δ5 Desaturase of *Thraustochytrium aureum*
TsΔ8D: Δ8-Sphingolipid desaturase of *Thalassiosira pseudonana*

## List of symbols

α: Alpha
γ: Gamma
Δ: Delta
#: Number
