# Supplementary material for: Isolation of a Δ5 Desaturase Gene from Euglena gracilis and Functional Dissection of Its HPGG and HDASH Motifs
Source: Lipids. 2012 Jun 24;47(9):913–26. doi: 10.1007/s11745-012-3690-1 (PMC3423564; doi:10.1007/s11745-012-3690-1)
Supplement: Supplementary file 1 — Supplementary material 1 (DOC 103 kb) [file 11745_2012_3690_MOESM1_ESM.doc]

**Electronic Supplemental Material**

**Table S1**. Primers used to amplify the Δ5 desaturase gene from *E. gracilis.*

| Primer Name | Sequence* |
| --- | --- |
| 5-1A | GGHCAYCAYRTBTAYACAAA |
| 5-1B | GGHCAYCAYRTBTAYACCAA |
| 5-1C | GGHCAYCAYRTBTAYACGAA |
| 5-1D | GGHCAYCAYRTBTAYACTAA |
| 5-5AR | TGRTGVACAAYYTGRWARTT |
| 5-5BR | TGRTGVACTAYYTGRWARTT |
| 5-5CR | TGRTGVACCAYYTGRWARTT |
| 5-5DR | TGRTGVACGAYYTGRWARTT |
| AUAP | GGCCACGCGTCGACTAGTAC |
| CDSIII 5’ | AAGCAGTGGTATCAACGCAGAGT |
| DNR CDS 5’ | CAACGCAGAGTGGCCATTACGG |
| ODMW469 | CTTCATCTTCCGGACCGCATTCTTGC |
| ODMWP479 | CTTAACCTTGCAGTTCTTGTCGGGGATC |
| ODMWP480 | CCGATACCAGTCAATTTGGTCAGCCTC |
| YL470 | CCTCACTGGGACCTCATTGCTGATCACC |
| YL791 | CACCTCGCGCATTGTCC-GCTTAACGTC |
| YL792 | TGCGAAGGGAGAATCATACGTGTAGAC |

*The nucleic acid degeneracy code used was as follows:

R= A/G; Y=C/T; W=A/T; B=G/T/C; V=G/A/C; and H=A/C/T.

**Fig. S1.** *Yarrowia* expression construct pDMW367-M4.

The construct pDMW367-M4 contains autonomous replication sequence 28 (40), a *URA3* gene (Genbank accession#: No. AJ306421) and a *FBAIN::EgΔ5D::Pex20* chimeric gene. The amino acid sequence of *EgΔ5D* in pDMW367 and pDMW367-M4 is identical, both having an arginine at position 347.

**
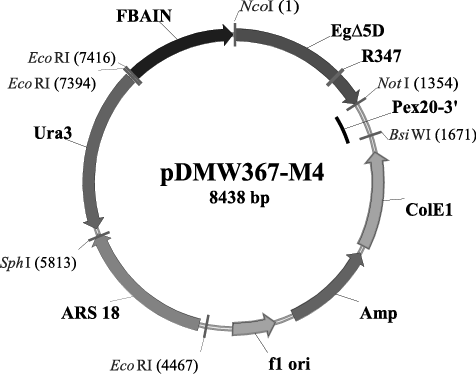
**

**Fig. S2.** HGLA and ARA distribution in FFA, PL and NL fractions.

**Quantification of Lipid Classes (Fig. S2)**

The analysis of different lipid classes was performed on oil samples extracted from yeast biomass using a modified Bligh and Dyer extraction described by Christie (Christie, W.W. (2003) *Lipid Analysis*, 3rd ed., The Oily Press, Bridgwater, U.K.). For each sample, biomass was incubated in 3 ml of 2:1 methanol/chloroform at room temperature for one hour with gentle agitation and inversion. 1 ml of chloroform and 1.8 ml of deionized water was then added; the mixture agitated and the chloroform phase was recovered after centrifugation. A second extraction was performed on the aqueous methanol layer with 1 ml of chloroform. The two extracts were combined and the solvent removed using a TurboVap™ at 50°C with dry nitrogen. The remaining oil was resuspended in an appropriate amount of 6:1 chloroform/methanol to obtain an approximate 100 mg/ml solution.

Lipid classes were separated by thin-layer chromatography (TLC) using pre-coated silica plates (silica gel 60; Merck EMD #5724-3), and lipid bands visualized using iodine vapor and identified by comparison to known standards (Sigma Aldrich, St. Louis, MO,USA). The lipid bands were isolated from the TLC plates, and fatty acid methyl esters prepared using 1 ml of a 1% methanolic hydrogen chloride solution with 15:0 TAG added as internal standard in a toluene co-solvent. Fatty acid methyl esters were extracted with 0.4 ml heptane after the addition of 1 ml 1M sodium chloride solution, and then separated by Agilent 7890A GC using an Omegawax column 30 m x 320 µm x 0.25 µm (Supelco Cat No. 24152) and helium carrier gas. The oven temperature was programmed from 160 °C to 240 °C at a rate of 10 °C/min with a final 4 min hold time. The proportion of each fatty acid was calculated based on the integrated peak area as calculated by Agilent ChemStation software relative to the area of the internal standard.
